# T-2 Toxin-Induced Oxidative Stress Leads to Imbalance of Mitochondrial Fission and Fusion to Activate Cellular Apoptosis in the Human Liver 7702 Cell Line

**DOI:** 10.3390/toxins12010043

**Published:** 2020-01-10

**Authors:** Junhua Yang, Wenbo Guo, Jianhua Wang, Xianli Yang, Zhiqi Zhang, Zhihui Zhao

**Affiliations:** Institute for Agri-Food Standards and Testing Technology, Shanghai Academy of Agricultural Sciences, Shanghai 201403, China; yangjunhua303@126.com (J.Y.); guo1103bo@126.com (W.G.); jianhuawang163@163.com (J.W.); yxl1218@126.com (X.Y.); zzqy@foxmail.com (Z.Z.)

**Keywords:** T-2 toxin, oxidative stress, mitochondrial fusion/fission, cellular apoptosis, HL-7702 cells

## Abstract

T-2 toxin, as a highly toxic mycotoxin to humans and animals, induces oxidative stress and apoptosis in various cells and tissues. Apoptosis and mitochondrial fusion/fission are two tightly interconnected processes that are crucial for maintaining physiological homeostasis. However, the role of mitochondrial fusion/fission in apoptosis of T-2 toxin remains unknown. Hence, we aimed to explore the putative role of mitochondrial fusion/fission on T-2 toxin induced apoptosis in normal human liver (HL-7702) cells. T-2 toxin treatment (0, 0.1, 1.0, or 10 μg/L) for 24 h caused decreased cell viability and ATP concentration and increased production of (ROS), as seen by a loss of mitochondrial membrane potential (∆Ψm) and increase in mitochondrial fragmentation. Subsequently, the mitochondrial dynamic imbalance was activated, evidenced by a dose-dependent decrease and increase in the protein expression of mitochondrial fusion (OPA1, Mfn1, and Mfn2) and fission (Drp1 and Fis1), respectively. Furthermore, the T-2 toxin promoted the release of cytochrome c from mitochondria to cytoplasm and induced cell apoptosis triggered by upregulation of Bax and Bax/Bcl-2 ratios, and further activated the caspase pathways. Taken together, these results indicate that altered mitochondrial dynamics induced by oxidative stress with T-2 toxin exposure likely contribute to mitochondrial injury and HL-7702 cell apoptosis.

## 1. Introduction

T-2 toxin, a fungal secondary metabolite that belongs to the type A trichothecene mycotoxin family, is produced by various *Fusarium* species [1]. T-2 toxin is a natural contaminant and commonly detected in cereals and feedstuff, and is typically present in northern temperate regions of America, Asia, and Europe [2]. As one of the most toxic trichothecene mycotoxins, T-2 toxin can cause serious toxicosis in humans and farm animals via ingestion of contaminated food and feed [3]. The fatal human disease alimentary toxic aleukia (ATA) and prevalence and development of the Kashin–Beck disease (KBD) are characterized by T-2 toxin exposure [4,5,6]. There is growing evidence to show that T-2 toxins could impair immunological functions; inhibit protein and DNA synthesis; promote oxidative stress; disturb energy balance and metabolism, cell cycle, and mitochondrial function; and induce cell apoptosis in vitro and in vivo [7,8,9].

During the past few decades, multiple studies suggested that T-2 toxin initiates the apoptotic process via oxidative stress, and then the cells subsequently enter the mitochondrial death pathway [10,11,12]. Some recent reports also indicated that mitochondrial toxicity is another major adverse effect of T-2 toxin, characterized by inhibition of enzymatic activity of mitochondrial respiratory chain (MRC) complexes, collapse of ∆Ψm, opening of the mitochondrial permeability transition pore (MPTP), decrease in intracellular adenosine triphosphate (ATP) contents, and increase in the level of reactive oxygen species (ROS), which could activate proapoptotic members of the B-cell lymphoma 2 (Bcl-2) family such as Bax and Bak, and subsequently trigger abnormal caspase-3 activity and apoptosis in different cell lines [11,13,14].

Known as the powerhouse of cells, mitochondria are dynamic organelles that continuously undergo fusion and fission (called “mitochondrial dynamics”), which is essential for maintaining physiological functions [15,16]. Fusion between mitochondrial outer membranes is mediated by mitofusin 1, 2 (Mfn1, Mfn2), whereas optic atrophy protein 1 (Opa1) participates only in the fusion of mitochondrial inner membranes [17]. Mitochondrial fission plays an important role in growing and dividing cells to populate them with adequate numbers of mitochondria, and the proteins that regulate mitochondrial fission mainly include mitochondrial fission 1 (Fis1) and dynamin related protein 1 (Drp1) [18]. These proteins not only determine the integrity of mitochondrial structure but also influence almost every aspect of mitochondrial function, such as the formation of ATP production, ROS, and respiration [19,20]. Apart from this, disorder of fusion/fission balance has been associated with the mechanism of cell apoptosis [21,22]. In discrete mitochondrial foci, Drp1 and Mfn2 have been shown to colocalize with Bax during the initial stages of apoptosis, which subsequently become mitochondrial scission sites by stimulation of hazard factors [21]. Additionally, induction of mitochondrial fission can advance cytochrome c release and active apoptosis, indicating that these fission proteins are an early event necessary for apoptotic progression [23,24]. Accumulated evidence suggests the intimate connection between mitochondrial dynamics and apoptosis induced by different exogenous toxic factors in various cells. A non-steroidal mycotoxin zearalenone (ZEA) is identified to trigger excessive mitochondrial fission by activation of the c-Jun N-terminal kinases/Drp1 pathway in endometrial stromal cells, resulting in structural and functional mitochondrial damage, release of cytochrome c into the cytoplasm, and subsequent induction of cellular apoptosis [25]. Simultaneously, ochratoxin A (OTA) exposure disturbs the balance of mitochondrial fusion/fission in GES-1 cells with a decrease in the expression of fusion proteins Mfn1 and Mfn2 [26]. Recent studies have shown that endocrine-disrupting chemicals such as benzo(a)pyren-7,8-dihydrodiol-9,10-epoxide (BPDE) and heavy metals like lead (Pb) or copper (Cu) can disturb the balance of mitochondrial fusion/fission dynamics and result in mitochondria-initiated apoptotic pathway, in vitro or in vivo [27,28,29]. However, the precise mechanism for T-2 toxin-induced apoptosis involved in mitochondrial dysfunction remains poorly understood.

In this study, we explore the potential effect of mitochondrial fusion/fission dynamics on T-2 toxin-induced hepatotoxicity using HL-7792 cell lines in vitro. We hoped that our finding would highlight the involvement of cellular oxidative stress, mitochondrial morphology, apoptotic cell death, and mitochondrial fusion/fission proteins in T-2 toxin-induced hepatotoxicity. These results should be helpful to explain the mechanism of mitochondrial fusion/fission involved in T-2 toxin-induced cellular apoptosis.

## 2. Results

### 2.1. Effect of T-2 Toxin on Changes in Cell Morphology and Viability

**Figure 1:**
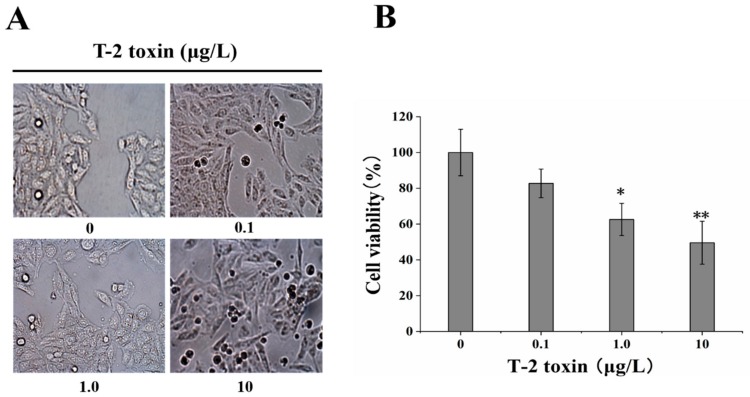
Cellular morphological changes and percentage of cell viability in HL-7702 cells exposed to 0, 0.1, 1.0, 10 μg/L T-2 toxin for 24 h. (**A**) micrographs of adherent cells (200 ×). The number of floating, dead cells were gradually increased along with the increase in the concentration of T-2 toxin; (**B**) cell viability, CCK-8 test results. Compared with control, the cell viability was decreased in different T-2 toxin-treated cells. Data are expressed as mean ± SEM from three different experiments. * *p* <0.05; ** *p* <0.01 vs. control values.

The morphology of HL-7702 cells treated with T-2 toxin was examined under light microscopy (Figure 1A). The cells in the control groups appeared long and spindle shaped and were densely packed. Furthermore, an increased number of floating cells in T-2 toxin-treated groups indicated the decrease of cell viability. Increasing T-2 toxin concentrations showed gradual rounding of the cells compared to the spindle-shape seen in the control group. In addition, cell counting kit-8 (CCK-8) assay kit was used to detect cell viability of HL-7702 cells in 0, 0.1, 1.0, and 10 μg/L T-2 toxin groups. HL-7702 cells showed higher viability in the absence of T-2 toxin, but the viability significantly decreased in response to T-2 toxin exposure with a dose-dependent manner (Figure 1B).

### 2.2. Effects of T-2 Toxin on the Generation of ATP and ROS

**Figure 2:**
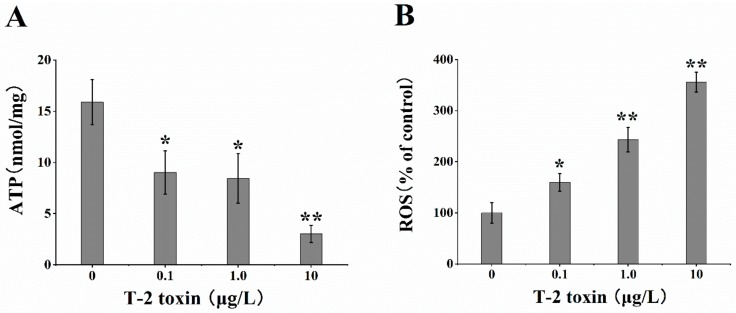
T-2 toxin exposure inhibited mitochondrial ATP level and exacerbated ROS generation in HL-7702. (**A**) Compared to the control group, the concentration of ATP was decreased, (**B**) while the level of ROS was increased in a dose-dependent manner after treatment with 0, 0.1, 1.0, and 10 μg/L T-2 toxin for 24 h. The average levels of ATP and ROS were expressed as mean ± SEM from three different experiments. * *p* <0.05; ** *p* <0.01 vs. control values.

After exposure to different levels of T-2 toxin for 24 h, the concentration of ATP and the level of ROS were detected. The results of ATP assay showed that T-2 toxin exposure led to the obvious decrease of intracellular ATP levels in a dose-dependent manner (Figure 2A). The concentration assay of ROS levels showed a significant increase by T-2 toxin treatment in a dose-dependent manner (Figure 2B).

### 2.3. Effects of T-2 Toxin on Mitochondrial Function

**Figure 3:**
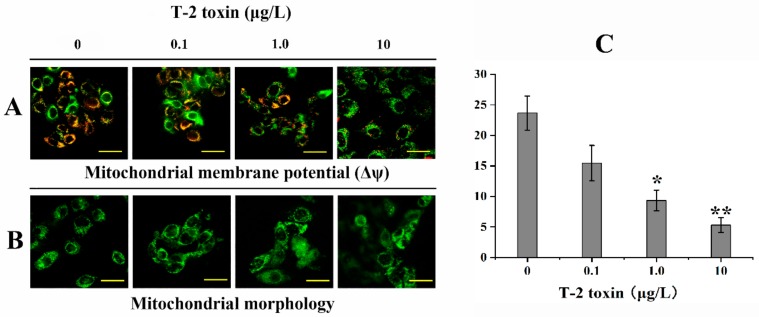
T-2 toxin exposure caused mitochondrial membrane potential (Δψm) collapse and mitochondrial fragmentation. The Δψm and mitochondrial morphology were respectively analyzed with the JC-1 kit and Mito-Tracker Green kit after treatment with 0, 0.1, 1.0, and 10 μg/L T-2 toxin for 24 h. (**A**) representative photomicrographs of cells in the JC-1 assay. JC-1 fluorescent dyes can gather in the mitochondrial matrix and produce red fluorescence. As the Δψm declines, JC-1 cannot accumulate in the matrix and hence exists in the matrix as a monomer, producing green fluorescence. (**B**) The ratio of the red (OD1) and green (OD2) optical density was markedly declined relative to the control. All values are presented as mean ± SEM from three different experiments. * p <0.05; ** p <0.01 vs. control values; (**C**) representative photomicrographs of mitochondrial morphology with Mito-Tracker Green assay. HL-7702 cells exposed to T-2 toxin exhibited significant mitochondrial fragmentation in a dose-dependent manner. Scale bar = 20 μm.

Mitochondrial membrane potential (Δψm) and mitochondrial morphology were directly used to clarify whether mitochondrial function was influenced by T-2 toxin exposure. Evaluation of ΔΨm was performed using the 5′,6,6′-tetrachloro-1,1′,3,3′-tetraethylbenzimidazolylcarbocyanine iodide (JC-1) dye in cells treated with different doses (0.1–10 μg/L) of T-2 toxin. T-2 toxin caused a decrease in red fluorescence, and an increase in green fluorescence with a dose-dependent manner (Figure 3A), indicating a downregulation in Δψm. Additionally, the fluorescence ratio of red to green was remarkably decreased in 1.0 and 10 μg/L T-2 toxin groups (Figure 3B). To establish whether T-2 toxin exposure was capable of inducing mitochondrial fragmentation and causing aberrations in mitochondrial dynamics, the HL-7702 cell was loaded with Mito Tracker Green after T-2 toxin exposure. The image presented a remarkable increase in mitochondrial fragmentation and an obvious swelling and elongation in mitochondrial structures (Figure 3C).

### 2.4. Effects of T-2 Toxin on Mitochondrial Dynamics-Related Genes and Proteins

**Figure 4:**
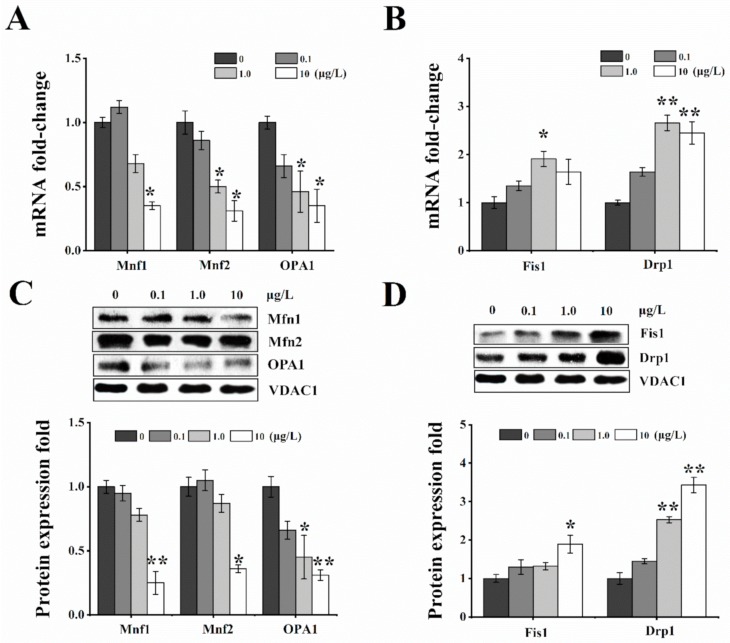
T-2 toxin exposure induced dynamic disorder of mitochondrial fusion/fission balance. The mRNA and protein expression of mitochondrial fusion (Mfn1, Mfn2, and OPA1) and fission (Fis1 and Drp1) were respectively evaluated by real-time PCR and Western blotting after treatment with 0, 0.1, 1.0, and 10 μg/L T-2 toxin for 24 h. (**A**) decrease in fusion related-gene levels of Mfn1, Mfn2, and OPA1 relative to control; (**B**) increase in fission related-gene levels of Fis1 and Drp1 relative to control; (**C**) decrease in protein levels of Mfn1, Mfn2, and OPA1 relative to control; (**D**) increase in protein levels of Fis1 and Drp1 relative to control. Voltage-dependent anion channel 1 (VDAC1) was used as a mitochondrial reference gene. All values are presented as mean ± SEM from three different experiments. * *p* <0.05; ** *p* <0.01 vs. control values.

The balance of mitochondrial dynamics is a principal procedure to determine cellular survival and apoptosis. Compared to the control, the mRNA expression levels of Mfn1, Mfn2, and OPA1 were significantly reduced in the 1.0 and 10 μg/L T-2 toxin groups (Figure 4A). However, the mRNA expression levels of Fis1 and Drp1 were remarkably increased in the same T-2 toxin dose groups (Figure 4B). The changes of mitochondrial fusion/fission proteins expression were corresponding to their respective mRNA level. The proteins expression of Mfn1, Mfn2, and OPA1 showed significant decrease in a 10 μg/L T-2 toxin group (Figure 4C), but the expressions of Fis1 and Drp1 proteins markedly increased in T-2 toxin groups with a dose-dependent manner, especially in the 1.0 and 10 μg/L toxin groups (Figure 4D). Taken together, T-2 toxin disturbs the dynamics balance of mitochondrial fusion and fission in HL-7702 cells and causes mitochondrial fragmentation.

### 2.5. T-2 Toxin Induced Cytochrome C Release into the Cell

**Figure 5:**
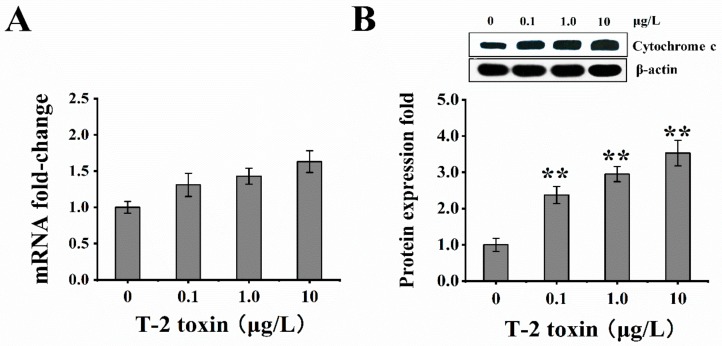
T-2 toxin exposure induced cytochrome c release from mitochondria to the cytosol. The mRNA and protein expression of cytochrome c were respectively evaluated after treatment with 0, 0.1, 1.0, and 10 μg/L T-2 toxin for 24 h. (**A**) increase in gene level of cytochrome c relative to control; (**B**) increase in protein level of cytochrome c relative to control. β-actin was used as a reference gene. All values are presented as mean ± SEM from three different experiments. * *p* <0.05; ** *p* <0.01 vs. control values.

Cellular cytochrome c release was used to assess whether the equilibrium of mitochondrial fusion/fission was disturbed and caused cellular apoptosis by exposure to T-2 toxin. The leakage of cytochrome c from the mitochondria to the cytosol was upregulated in T-2 toxin groups; notably, the protein expression of cytochrome c was also observably higher in T-2 toxin groups (Figure 5A,B).

### 2.6. Effects of T-2 Toxin on Mitochondrial-Mediated Cellular Apoptosis

**Figure 6:**
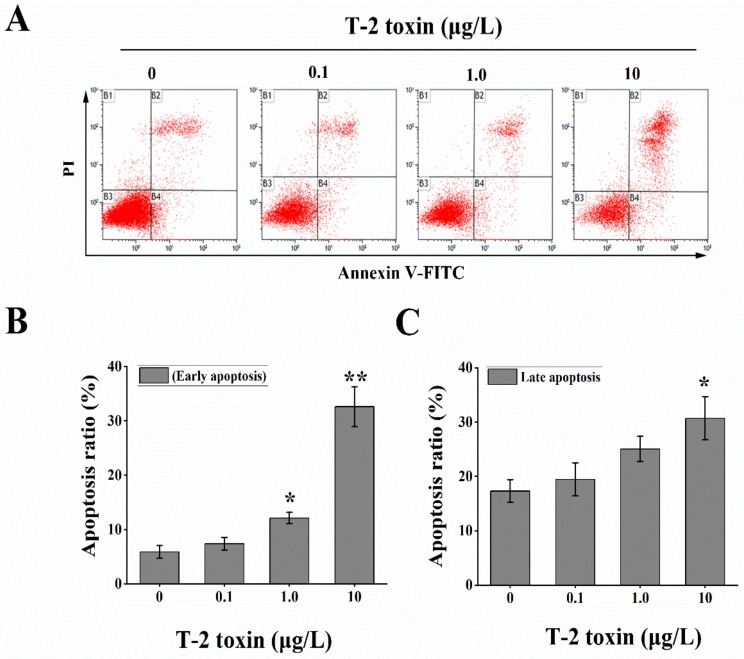
T-2 toxin exposure increased the cellular apoptosis. (**A**) the apoptotic ratio was evaluated by Annexin V-FITC/PI assay after treatment with 0, 0.1, 1.0, and 10 μg/L T-2 toxin for 24 h; (**B,C**) T-2 toxin treatment increased the early and late apoptosis ratio. All values are presented as mean ± SEM from three different experiments. * *p* <0.05; ** *p* <0.01 vs. control values.

To determine the effects of T-2 toxin on HL-7702 cellular apoptosis, we tested the ratio of early and late apoptosis with the Annexin V-Fluorescein isothiocyanate (FITC)/propidium iodide (PI) assay kit (Figure 6A). After exposure to different levels of T-2 toxin for 24 h, the cells treated with 1.0 and 10 μg/L T-2 toxin showed higher rates of not only the early apoptosis (12.1% and 32.6% vs. 5.86% in the control group, respectively) (Figure 6B), but also late apoptosis (25.06% and 30.72% vs. 17.31% in the control group, respectively) (Figure 6C).

**Figure 7:**
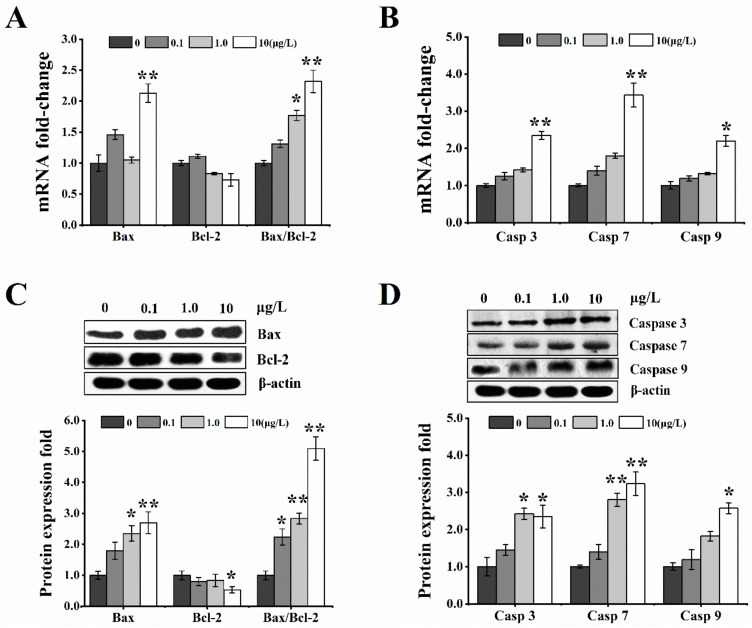
T-2 toxin exposure induced increase in the apoptosis-related protein expression by mitochondria-mediated pathway in HL-7702 cells. The mRNA and protein expressions of apoptosis-related proteins (Bax, Bcl-2, caspase 3, caspase 7, and caspase 9) were respectively evaluated after treatment with 0, 0.1, 1.0, and 10 μg/L T-2 toxin for 24 h. (**A**) increase and decrease in the gene levels of Bax and Bcl-2, respectively, relative to control; (**B**) increase in gene levels of caspase 3, caspase 7, and caspase 9 relative to control; (**C**) increase and decrease in the protein levels of Bax and Bcl-2, respectively, relative to control; (**D**) increase of protein levels of caspase 3, caspase 7, and caspase 9 relative to control. β-actin was used as a reference gene. All values are presented mean ± SEM from three different experiments. * *p* <0.05; ** *p* <0.01 vs. control values.

Following real-time PCR and Western blot methods, the expressions of Bax and Bcl-2 were examined in HL-7702 cells exposed to different levels of T-2 toxin. The mRNA level of Bax was considerably higher in the 10 μg/L dose group, but was only slightly increased in the 0.1 and 1.0 μg/L toxin dose groups. Furthermore, the mRNA level of Bcl-2 in all T-2-treated groups showed a slight decrease, but the ratio of Bax/Bcl-2 was remarkably higher in 1.0 and 10 μg/L T-2 toxin-treated groups (Figure 7A). Simultaneously, the change of Bax and Bcl-2 proteins expressions was corresponding to their respective mRNAs levels; the results presented a notable increase in the protein level of Bax in all T-2 toxin groups in a dose-dependent manner, and an obvious downregulation in protein expression of Bcl-2 at 10 μg/L, which led to an increase in the ratio of Bax/Bcl-2 (Figure 7C).

In addition, the mRNA and protein expressions of caspase 3/7/9 showed a similar pattern. The results showed not only significant upregulation in the mRNA levels of caspase 3/7/9 at 10 μg/L (Figure 7B), but also remarkable increases in their respective protein expressions at 1.0 and 10 μg/L toxin doses (Figure 7D). Taken together, T-2 toxin exposed to HL-7702 cell for 24 h could induce cellular apoptosis.

## 3. Discussion

Our data support the idea that cellular apoptosis after T-2 toxin exposure is a consequence of abnormal mitochondrial morphology and mitochondrial fusion/fission imbalance. T-2 toxin-enhanced mitochondrial-dependent apoptotic pathway has been found in various cell lines and tissues [11,30,31]. On the basis of these studies, we observed that T-2 toxin triggered oxidative stress, disrupted mitochondrial fusion/fission balance, caused Δψm collapse, decreased fusion-related proteins expression, and increased fission-related proteins expression, leading to cytochrome c release, pro-apoptotic factors’ induction, and ultimately cellular apoptosis. These results show that a T-2 toxin triggers oxidative stress and interrupts the dynamic balance between mitochondrial fusion and fission, with this equilibrium being essential for a wide range of biological processes.

After consumption of T-2 toxin-contaminated food and feed, it was easy to cause severe diseases or health risks in humans or animals [32]. Previously, research showed that T-2 toxin could lead to cytotoxicity in various cell types. In our experiment, the cells exposed to T-2 toxin upper 0.1 μg/L showed dose-dependent cytotoxic effects, such as the increase in intercellular spaces, alteration in morphology, and decrease in viability. Quantitative cell viability assays using CCK-8 showed that T-2 toxin inhibited proliferation of HL-7702 cells in a dose-dependent manner after a 24 h exposure and exhibited cytotoxic response. The present result was consistent with other previous studies [33,34,35].

Oxidative stress exerts a pivotal role in toxicity of the T-2 toxin [9,13]. Overproduction of ROS or decreased cellular antioxidant are typical features of oxidative stress [36,37]. In the current study, it was clear that elevated T-2 toxin concentration could result in excessive ROS production and cause injury to HL-7702 cells. This was consistent with other information that T-2 toxin exposure could induce ROS production in Bel-7402, Chang liver, and HeLa cell lines [9]. Similar results were also found in broiler hepatocytes treated with T-2/HT-2 toxins [38], which resulted in ROS accumulation in chicken primary hepatocytes. In vivo, the ROS level in the mice brain tissue showed a significant increase after T-2 toxin exposure, and the upregulation of antioxidant enzymes expression was also observed in broiler liver and mice brain tissue [38,39], respectively. Therefore, it is reasonable to believe that oxidative stress plays a critical role in the cellular apoptosis induced by T-2 toxin.

Under normal physiological conditions, mitochondria are the main source of ROS production. Understandably, the production and elimination of ROS should keep in the balance, as sustained ROS production could make the mitochondria become the main target of ROS [40]. Literature studies on different cell lines have indicated that T-2 toxin exposure cause severe mitochondrial dysfunction due to lipid peroxidation and ROS production, including inhibition of mitochondrial respiratory chain complex, mitochondrial transport system, exacerbation of oxidative phosphorylation, and loss of Δψm [13]. In addition, as the Δψm provides a driving force for energy production, decrease in Δψm disturbs the coupling efficiency between phosphorylation and oxidation, resulting in large bioenergetic deficits. In the present study, Δψm reduced in a dose-response manner after T-2 toxin exposure. Simultaneously, the abnormalities of mitochondrial shape were also observed, including membrane fragmentation and swelling. Mitochondria showed more serious extent of rupture and dissolution with increasing T-2 toxin doses. One research reported that exposure to T-2 toxin reduced Δψm and ATP levels and increased intracellular ROS generation [13]. Similarly, the loss of Δψm and inhibition of ATP-linked oxygen consumption rates were detected in murine embryonic stem cells and rat cardiomyocytes that underwent T-2 toxin treatment [11,31]. In the pituitary tumor cell line GH3, T-2 toxin at the dose of 10 and 40 nM could significantly decrease the Δψm by 19.85% and 47.49% after 24 h of treatment, respectively [41]. Our results found that exposure of T-2 toxin in HL-7702 significantly decreased Δψm and ATP generation in a dose-response manner. This study evidenced that T-2 toxin could inhibit mitochondrial dysfunction and promote mitochondria fragmentation accompanied by deficiency of ATP supply and oxidative stress, which could greatly contribute to the disorder of mitochondrial dynamic balance.

Mitochondria are dynamic organelles that undergo continuous fission and fusion to maintain interconnected networks or small individual units to rapidly respond to environmental stimuli or damages. For this reason, the balance of mitochondrial fission and fusion is pivotal for cellular homeostasis, which is involved in the stable expression of mitochondrial fission/fusion-related proteins. The reduced expression of Mfn1/2 or OPA1 proteins and increased expression of Drp1 protein could cause the inhibition of mitochondrial fusion and mitochondrial fragmentation [42]. Human lung cancer cell lines or lung tumor tissue samples all exhibit an imbalance of Drp1/Mfn2 expression, which facilitates a state of mitochondrial fission. However, overexpression of Mfn2, Drp1 knockdown, or Drp1 inhibition in lung cancer cells could restore mitochondrial network formation and result in an obvious increase in spontaneous apoptosis and reduction of cancer cell proliferation [43,44]. Additionally, Drp1 is one kind of GTPase protein that affects the mitochondrial morphology and plays an important role in peroxisomal fission. Drp1 homozygote knockout mice (Drp1-/-) have developmental abnormalities [45], and Drp1 knockout human colon cancer HCT116 cells display an elongated mitochondrial morphology and fragmentation [46]. However, studies on cellular dysfunctions and mitochondrial fusion/fission imbalance are rather scarce and limited to OTA, phomoxanthone A (PXA), and ZEA [25,26,47]. Human gastric epithelium cells (GES-1) treated with 5μM, 10μM, and 20 μM OTA showed a decrease in Drp1, Mfn2, and OPA1 expressions that suggest that OTA breaks mitochondrial dynamics balance [26]. Similarly, in endometrial stromal cells (ESCs), ZEA treatment triggered excessive mitochondrial fission with increase in the Mito-Drp1 protein expression, thereby resulting in structural and functional damage of mitochondrial and subsequently induced the mitochondrial apoptosis pathway [25]. Our results exhibited the same pattern as observed with ZEA, in that the expression of Mfn1, Mfn2, and OPA1 were decreased by T-2 toxin treatment, whereas those of the fission-related proteins Drp1 and Fis1 upregulated. These results imply that the increased fission and decreased fusion lead to the mitochondrial membrane fragmentation, collapse of Δψm, and release of cytochrome c from the mitochondria to the cytosol, which was supported by results of the cytochrome c assay.

In this study, T-2 toxin exposure caused an increase in the expression of cytochrome c in the cytoplasm. Fang et al. [11] indicated that embryonic stem (ES) cells cultivated with T-2 toxin at 0.5, 1, and 2 ng/mL dose showed loss of Δψm, resulting in the opening of MPTP and release of cytochrome c from the mitochondria intermembrane space to the cytosol. Our results were in line with previous reports that T-2 toxin (10 and 20 ng/mL) upregulated the expression of cytochrome c in the cytoplasm of human chondrocytes [13].

The dynamic balance of mitochondria fusion/fission is very crucial for many biological processes, and aberrations in mitochondrial dynamics may induce the initiation of the cellular apoptosis pathway [48]. In this pathway, in the upstream process of Δψm, the Bcl-2 family is crucial to the opening of MPTP and release of cytochrome c, and the downstream process is involved in the activation of the caspase pathway [49]. In the Bcl-2 family, the anti-apoptotic protein Bcl-2 and pro-apoptotic proteins Bax and Bak, all of which play crucial roles in cellular apoptosis. It was found that the expression of Bax is increased and that of Bcl-2 is decreased in the differentiated ES cells treated with T-2 toxin [11]. Similar apoptotic results were reported for T-2 toxin exposure in neuroblastoma-2a cells, human chondrocytes, primary hepatocytes of broilers, and male mice germ cells [30,50,51]. Our results confirmed that T-2 toxin induced apoptosis via the mitochondrial apoptotic pathway in HL-7702 cells. Furthermore, the increased expression of Bax and the decrease expression of Bcl-2 were detected and the ratio of Bax/Bcl-2 also improved. Both Bax and Bak have been demonstrated to co-localize with Mfn2 in the outer mitochondrial membrane (OMM) [21,52]. Under the pressure of exogenous stimulus, Bax was shown to bind to Mfn2 and inhibit its pro-fusion function [52], which was found to decrease the mitochondrial membrane stability, thereby facilitating Drp1-mediated mitochondrial fission, and promoting the formation of hemifusion-related hole and leakage of cytochrome c [53]. Whelan et al. [54] also found that Bax/Bak knockout mice showed a resistance to MPTP opening in cardiac myocytes, suggesting that Bax plays distinct roles in regulating apoptosis. These findings suggest that T-2 toxin induces HL-7702 cell apoptosis through upregulation of Bax expression and combined with mitochondrial fusion/fission imbalance, triggered as a consequence of cytochrome c release.

Downstream of the mitochondrial dynamic imbalance, cytosolic cytochrome c activates the caspase cascade including both execution of caspase-3 and initiation of the caspase-9 [11]. In our experiment, T-2 toxin exposed to HL-7702 cells remarkably increased the expression of caspase 3, 7, and 9 mRNA and proteins in a dose-response manner. In neuroblastoma-2a cells, T-2 toxin induced the increase of caspase 3, 7, and 9 protein expressions in a dose-dependent manner [50]. Additionally, the T-2 toxin has been proved to induce apoptosis in various cell types such as Vero, human chondrocytes, Bel-7402, HeLa, HL-60, U937 cells, and human liver cells in vitro, all of which is closely related to Bcl-2, Bax, and caspase 3 and 9 signaling pathways [9,55]. These results showed that T-2 toxin exposure to HL-7702 cells triggered apoptosis. There is no strict association between cellular apoptosis and mitochondrial fission/fusion, but alteration of the mitochondrial morphology reportedly modifies the ability of Bax to permeabilize the OMM [56]. In addition, Drp1^-/-^ mice displayed defects in neural tube closure and was demonstrated to be associated with cellular apoptosis [57], while extensive fission inhibited endoplasmic reticulum stress-mediated apoptosis in Mfn1-deficient cells, and restoration of Δψm by inhibition of Drp1 facilitated cell death [56]. This is consistent with the report that the fission could not trigger apoptosis, which depends on the activity of pro-apoptotic factors, resulting from the remodeling of mitochondrial morphology to efficiently facilitate apoptosis [58]. Therefore, we could speculate that mitochondrial dynamic imbalance inducing fission is likely an integral step during the apoptotic procedure, which depends on the activity of pro-apoptotic factors to efficiently promote apoptosis. This is consistent with the consequence of impaired fission on apoptosis being context-dependent [59]. However, further research is needed to support this hypothesis.

## 4. Materials and Methods

### 4.1. Chemicals

HL-7702 cells were provided by Shanghai Huiying Biological Technology Co., Ltd (Shanghai, China). T-2 toxin was purchased from Beijing Puhuaren Technology Development Co., Ltd (Beijing, China). Dulbecco’s modified eagle medium (DMEM), fetal bovine serum (FBS), collagenase type IV, and penicillin–streptomycin was purchased from Gibco (Thermo Fisher, Shanghai, China). Cell Counting Kit-8 (CCK-8), Annexin V-FITC/PI apoptosis detection kit, ATP assay kit, ROS assay kit, and mitochondrial membrane potential assay kit with JC-1 were purchased from Beyotime Biotechnology (Beyotime, Haimen, China). All real-time PCR reagents were purchased from TaKaRa (Dalian, China). All other chemicals, if not stated, were purchased from Sigma.

### 4.2. Cell Culture

HL-7702 cells were cultured in DMEM supplemented with 10% (*v*/*v*) heat-inactivated FBS and 100 units/mL penicillin and 100 μg/mL streptomycin at 37 °C in a humidified 5% CO_2_ incubator. T-2 toxin was dissolved in dimethyl sulfoxide (DMSO) [the final concentration of DMSO was controlled below 0.1% (*v*/*v*) and had no adverse effect on the cellular parameters)] and diluted with cell culture solution.

### 4.3. Measurement of Cell Viability

Cell viability was determined using the CCK-8 kit. In brief, HL-7702 cells were seeded into 96-well culture plates (1 × 10^4^ cells/well). After 12 h culture, these cells were added with different levels of T-2 toxin (0, 0.1, 1.0, and 10 μg/L) for 24 h. In control, the cells were incubated with the vehicle 0.1% DMSO in DMEM. Subsequently, changes in cellular growth and morphology were observed by inverted light microscopy (Olympus IX71, Hamburg, Germany). Then, the medium was discarded and supplied by 100 μL DMEM containing 20 μL CCK-8. The absorbance was recorded using the microplate reader iMark (Bio-Rad Laboratories, Inc., Hercules, CA, USA), and the values were normalized to the untreated cells. A blank control group (no cells) was set up and six replicates were performed in each group.

### 4.4. Measurement of Cellular ATP Level and ROS Generation

Cellular ATP level was determined with the ATP-lite kit. HL-7702 cells at a density of 1 × 10^4^ cells were seeded into black opaque 96-well plates with T-2 toxin at final concentrations of 0, 0.1, 1.0, and 10 μg/L for 24 h, respectively. ATP substrate was added into each well (1:1, v/v) in the dark. Plates were mixed for 5 min, and luminescence intensity was measured by automatic chemiluminescence immunoassay analyzer (Maccura Biotechnology, Chengdu, China). Cellular ROS generation was detected using the 2′,7′-dichlorodihydrofluorescein diacetate (DCFH-DA) kit. In brief, HL-7702 cells with 5 × 10^5^ were seeded into 6-well plates and incubated with T-2 toxin (0, 0.1, 1.0, and 10 μg/L) for 24 h. After exposure, DCFH-DA (10 μM) was added and incubated at 37 °C for 30 min in the dark. After washing, the cells were harvested by trypsinization, and assayed for ROS production by using a microplate reader.

### 4.5. Measurement of Mitochondrial Membrane Potential (∆ψm) and Mitochondrial Morphology

A mitochondrial membrane potential assay kit with JC-1 was utilized to determine the ∆Ψm within fluoride-treated cells. The HL-7702 cells were incubated with 0, 0.1, 1.0, and 10 μg/L T-2 toxin for 24 h. After exposure, the cells were collected by trypsinization, re-centrifuged, and finally resuspended in complete DMEM at the concentration of 1 × 10^5^ cells/mL. The cells were kept in the 5% CO_2_ incubator for 20 min after being mixed with the 1 mL JC-1 stain liquid. After washing, the cells were imaged by using a fluorescence microscope. Mitochondrial morphology was evaluated by staining with Mito-Tracker Green (Beyotime Biotechnology, Haimen, China). After 24 h treatment, the HL-7702 cells were washed and stained for 0.5 h with Mito-Tracker Green; then, the images were captured (Leica SP5, Leica Microsystems Inc., Wetzlar, Germany).

### 4.6. Measurement of Cellular Apoptosis

Cellular apoptosis was performed using an Annexin V-FITC/PI Cell apoptosis assay kit. In brief, the HL-7702 cells were treated with T-2 toxin at final concentrations of 0, 0.1, 1.0, and 10 μg/L for 24 h. The HL-7702 cells in control group were incubated with the vehicle, i.e., 0.1% DMSO in DMEM. After centrifugation and resuspension in staining buffer, the cells were incubated in Annexin V-FITC and PI solution for 20 min at room temperature. Fluorescence signal was immediately detected by flow cytometry (Beckman Coulter, Gallios, Indianapolis, IN, USA), and data were analyzed using CXP analysis software version 2.2 (Beckman Coulter Inc., Brea, CA, USA).

### 4.7. RNA Extraction and Real-Time Quantitative PCR

The mRNA expression levels for the dynamin and caspase family members in HL-7702 cells were determined by qPCR. Total RNA was extracted using RNAiso Plus and cDNAs were prepared using a First Strand cDNA Synthesis Kit.

The expression of different genes was quantified using ABI-prism 7500 Sequence Detection System (Applied Biosystems, Inc., Foster City, CA, USA). The levels of genes in the mitochondria were normalized to VDAC1 as the reference gene, while that of other genes were normalized to β-actin. As shown in Table 1, the primer sequences for Mfn1, Mfn2, OPA1, Fis1, Drp1, VDAC1, caspase 3/7/9, cytochrome c, Bax, Bcl-2, and β-actin were designed and synthesized by Shanghai Generay Biotech Co., Ltd. (Shanghai, China). QPCR reaction was performed according to the manufacturer’s instructions. Then, the 2^−ΔΔCt^ method was used to analyze the relative expression of each gene [60].

### 4.8. Protein Extraction and Western Blotting Analysis

After washing, HL-7702 cells were lysed with radioimmunoprecipitation assay buffer (RIPA) lysis buffer on ice. The lysates were further disrupted by sonication and were centrifuged for 20 min at 13000 g and 4 °C.

In addition, 30 μg protein was separated in 7.5, 10, and 15% sodium dodecyl sulfate polyacrylamide gel electrophoresis (SDS-PAGE), respectively. The separated proteins were transferred onto a nitrocellulose (NC) membrane by a Bio-Rad tank blotting system. Then, membranes were probed with caspase 3 (35-1600Z, Thermo Scientific, 1:200), caspase 7 (MA1-16839, Thermo Scientific, 1:700), caspase 9 (MA1-12562, Thermo Scientific, 1:1000,), cytochrome c [1:2000, ab133504,], Bax (ab182734, 1:1000), Bcl-2 (14-1028-82, Thermo Scientific, 1:500), Mfn1 (ab129154, 1:5000), Mfn2 (ab56889, 1:2000), OPA1 (ab157457, 1:2000), Fis1 (ab96764, 1:1000), Drp1 (ab184248, 1:2000), β-actin antibody (MA5-15739, Thermo Scientific, 1:3000), or VDAC1 (ab154856, 1:2000) in tris buffered saline with Tween-20 (TBST) at 4 °C overnight. After that, the blots were incubated with horseradish peroxidase (HRP)-conjugated goat anti-rabbit IgG (A32731, Thermo Scientific, 1:20000) or HRP-conjugated goat anti-mouse IgG (A32723, Thermo Scientific, 1:10000). Immunoblots were determined with enhanced chemiluminescence. The photographs were recorded and analyzed by a Bio-Rad ChemiDoc Touch Imaging System. The band density of cellular proteins was normalized to β-actin, while those of the mitochondrial proteins was normalized to VDAC1. All the antibodies labelled with Thermo Scientific were provided by Thermo Fisher Scientific-CN (Shanghai, China), others were purchased from Abcam (Shanghai) Trading Co., Ltd (Shanghai, China).

### 4.9. Statistical Analysis

All analyses were used SPSS 16.0 (SPSS Inc., Chicago, IL, USA). Statistical analyses were performed by one-way ANOVA, followed by LSD’s post-hoc test. All of the data represent the mean ± SEM, and *p* ≤0.05 were considered statistically significant.

## 5. Conclusions

T-2 toxin exposure contributed to an increase in ROS generation and a decrease in ATP levels. The oxidative stress further increased mitochondrial membrane potential and destroyed mitochondrial morphology. Damage to the mitochondrial shapes were likely due to a decrease in expression of fusion proteins and an increase in those of fission proteins, resulting in mitochondrial fragmentation and dysfunction. Subsequently, a decrease in anti-apoptotic proteins and an increase in pro-apoptotic were detected, wherein cytochrome c leaked out from the mitochondria to the cytoplasm, triggering caspase activation and inducing cellular apoptosis. These data indicate that the underlying mechanism of T-2 toxin-induced cellular apoptosis contributes to dynamic imbalance of mitochondrial fusion/fission triggered by oxidative stress, which leads to mitochondrial fragmentation and facilitates Bax-dependent cytochrome c release following apoptotic signals.

## Figures and Tables

**Table 1:** Primer sequences for qPCR.

Title	GenBank Accession Number	5’–3’ Primer Sequence	PCR Product/bp
*Caspase 3*	NM_004346.3	F: atgctgaaacagtatgccgacaa	98
		R: gcgtcaaaggaaaaggactcaaat	
*Caspase 7*	BC015799.1	F: tgacttcctcttcgcctattccac	124
		R: gatttccaggtcttttccgtgc	
*Caspase 9*	NM_001229.4	F: agccaaccctagaaaaccttacc	115
		R: tcaccaaatcctccagaaccaat	
*Bax*	NM_004324.3	F: ccccgagaggtctttttccgag	113
		R: agggccttgagcaccagtttg	
*Bcl-2*	BC027258.1	F: cctctgtttgatttctcctggc	153
		R: ttctactgctttagtgaaccttttg	
*cytochrome c*	NM_018947.5	F: cttcagaaataaggaaatagggga	154
		R: caaaataagcatgtaggtggca	
*Fis1*	NM_016068.2	F: gcacgcagtttgagtacgcct	113
		R: ctgttcctccttgctccctttg	
*Mfn1*	NM_033540.2	F: ttgagagatgacctggtgttagtag	130
		R: ttagtgttgattcagagtttgcgac	
*Mfn2*	BC017061.1	F: tctttatgctgatgttgagttttgg	159
		R: tttgggagaggtgttgcttattt	
*Drp1*	BC024590.1	F: gcatcacatcagagattgtttacca	113
		R: tagcacttttatcatccacgggtt	
*OPA1*	BC075805.1	F: aatgactttgcggaggacagc	145
		R: ttgcattttcagtatccttgagacg	
*VDAC1*	NM_003374	F: accgagattactgtggaaga	75
		R: agtgttaggtgagaaggatga	
*β-actin*	NM_001101.3	F: gcatgggtcagaaggattcc	276
		R: tggatagcaacgtacatggc	
